# The BP_3_ monolayer as a high-capacity and rapid-diffusion anode for sodium-ion batteries: a first-principles study

**DOI:** 10.1039/d5na00736d

**Published:** 2025-11-28

**Authors:** Tuan V. Vu, Duc-Quang Hoang, Thi H. Ho, Hoang Van Chi, Khang D. Pham

**Affiliations:** a Laboratory for Computational Physics, Institute for Computational Science and Artificial Intelligence, Van Lang University Ho Chi Minh City Vietnam tuan.vu@vlu.edu.vn; b Faculty of Mechanical, Electrical, and Computer Engineering, Van Lang School of Technology, Van Lang University Ho Chi Minh City Vietnam; c Faculty of Applied Sciences, HCMC University of Technology & Education 01 Vo Van Ngan, Thu Duc Ho Chi Minh City 700000 Vietnam; d Department of Scientific Research Management, 108 Military Central Hospital Hanoi 100000 Vietnam; e Institute of Research and Development, Duy Tan University Da Nang 550000 Vietnam phamdinhkhang@duytan.edu.vn; f School of Engineering & Technology, Duy Tan University Da Nang 550000 Vietnam

## Abstract

The rapid development of sodium-ion batteries (SIBs) as a cost-effective alternative to lithium-ion technology demands the discovery of high-performance anode materials with large capacity, good stability, and fast ion transport. In this work, we perform a comprehensive first-principles study to evaluate the potential of the BP_3_ monolayer as an anode material for SIBs. Our results show that the material exhibits excellent mechanical stability, intrinsic metallic behavior, and strong affinity toward Na-ion adsorption. In addition, Na ions diffuse on the BP_3_ monolayer with a low migration barrier of 0.13 eV, suggesting fast charge/discharge kinetics. Upon full sodiation, the system retains its metallic conductivity, which is essential for efficient electron transport. The open-circuit voltage remains within a practical range during Na insertion, with an average value of 0.27 V. In particular, a theoretical storage capacity of 2325.58 mAh g^−1^ is obtained, which is higher than that of many previously reported 2D anode materials. These findings highlight the BP_3_ monolayer as a promising anode material for next-generation high-capacity and fast-charging sodium-ion batteries.

## Abstract 2

BP_3_ monolayer as a high-performance anode for sodium-ion batteries, featuring ultrahigh Na storage capacity, fast ion diffusion, and a stable sodiation voltage.

## Graphical abstract

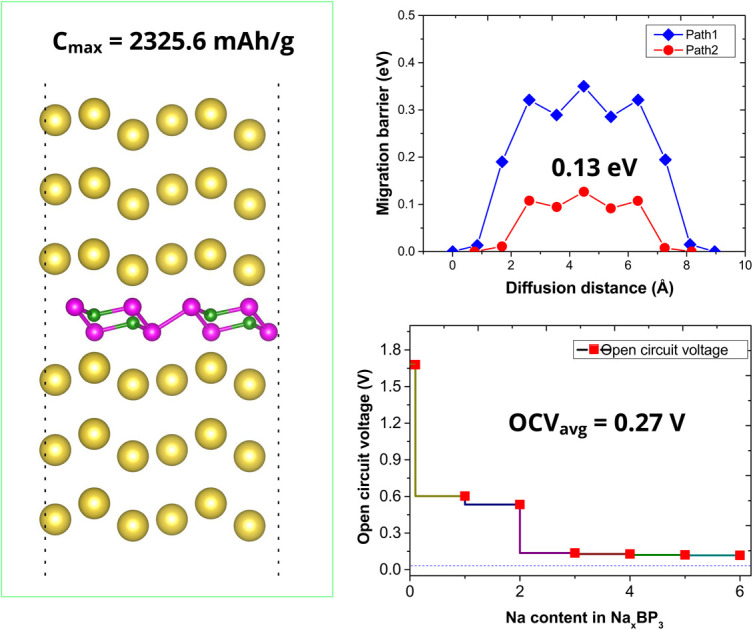

## 1 Introduction

Lithium-ion batteries (LIBs) have become the foundation of modern energy storage technologies, widely used in portable electronics, electric vehicles, and renewable energy systems, due to their high energy density, long cycle life, and relatively mature commercial infrastructure.^1,2^ However, the increasing global demand for energy storage, particularly at a large scale, has revealed several critical limitations of LIBs. These include the scarcity and uneven geographic distribution of lithium resources, rising production costs, and safety concerns related to thermal runaway and flammable electrolytes.^3,4^ These issues not only threaten the sustainability of LIB technology but also drive the urgent search for alternative battery systems that are more cost-effective, safer, and based on more abundant materials.

Sodium-ion batteries (SIBs) have emerged as a promising next-generation energy storage technology that could complement or even replace LIBs in specific applications. Sodium, being the fourth most abundant element in the Earth's crust, is inexpensive and widely available across the globe. Moreover, sodium shares similar electrochemical behavior with lithium, enabling the adaptation of many existing LIB design principles to SIBs.^5,6^ Although the larger ionic radius and heavier atomic mass of Na^+^ compared to Li^+^ pose some challenges in terms of diffusion and volume change, SIBs still show great potential, particularly in large-scale energy storage systems (ESS), where cost and resource accessibility outweigh the size and weight concerns.^7^ In addition, when contrasted with all-solid-state batteries,^8,9^ SIBs benefit from the relatively simpler manufacturing routes and reduced material costs while still maintaining competitive electrochemical performance. These advantages collectively highlight the potential of SIBs as a cost-effective and scalable solution for next-generation rechargeable batteries.

As in all rechargeable batteries, the choice of electrode materials plays a decisive role in determining the overall performance of SIBs. In particular, the anode material critically influences the specific capacity, rate capability, and cycling stability of the battery.^10,11^ To be effective, a sodium-ion anode must exhibit strong Na-ion storage capability, fast Na-ion diffusion, good electrical conductivity, low working voltage *versus* Na/Na^+^, and robust structural integrity during repeated charge/discharge processes.^12^ To date, a variety of anode materials for SIBs have been explored, including hard carbon, transition metal oxides, sulfides, phosphides, and alloy-based materials.^13^ While many of these systems exhibit acceptable capacity and cyclability, challenges such as poor rate performance, structural degradation, and large volume expansion still remain. In recent years, two-dimensional (2D) materials have attracted significant attention as potential anodes due to their large surface area, flexible layered structures, and highly tunable physicochemical properties, all of which are favorable for enhancing Na-ion adsorption, diffusion, and reversibility.^14,15^ These unique advantages make 2D materials promising candidates for achieving fast Na^+^ diffusion kinetics, high storage capacities, and stable electrochemical reversibility in SIBs.

In recent years, 2D boron phosphide (BP)-based materials and their derivatives such as BP_2_ and B_3_P have attracted considerable attention due to their outstanding potential as anode candidates for alkali metal batteries (Li, Na, and K). Jiang *et al.*^16^ also revealed that the pristine BP monolayer exhibits strong adsorption for Li, Na, and K atoms without requiring high energy barriers and undergoes a semiconductor-to-metal transition upon ion adsorption, thereby ensuring electrical conductivity. Notably, the material features low diffusion barriers (0.217 eV for Na and 0.155 eV for K) and impressive storage capacities (1283 mAh g^−1^ for Li and 570 mAh g^−1^ for K), further reinforcing its promise as an anode material. In addition, Ye *et al.*^17^ demonstrated that the BP_2_ monolayer possesses intrinsic metallicity, which enables efficient electron transport and offers an ultralow Na diffusion barrier of only 0.03 eV, along with high theoretical capacities of 368.5 mAh g^−1^ for Na and 737.0 mAh g^−1^ for Li. In another study, Abbas *et al.*^18^ established that the B_3_P monolayer exhibits excellent dynamical, mechanical, and thermal stability, while retaining metallicity upon ion insertion. Specifically, a high theoretical capacity of 1691 mAh g^−1^ was achieved, and the low migration barriers (0.370 eV for Li and 0.156 eV for Na) indicate fast ion transport and excellent rate capability. Beyond alkali-ion batteries, Yu *et al.*^19^ proposed the BP monolayer as an effective anchoring material in lithium–sulfur (Li–S) batteries due to its moderate binding energies with polysulfides, which enhance the electronic conductivity and suppress the shuttle effect. These findings collectively highlight the 2D boron phosphide family as highly promising candidates for next-generation battery anodes, owing to their structural stability, excellent electrical conductivity, high ion storage capacities, and favorable ion diffusion kinetics.

In this study, we conduct a comprehensive first-principles investigation of the electrochemical properties of the BP_3_ monolayer as a potential anode material for SIBs. Using density functional theory (DFT) calculations, we examine its structural stability, Na adsorption energies, electronic structure evolution upon sodiation, charge transfer characteristics, Na-ion diffusion barriers, and theoretical specific capacity. Our results provide key insights into the feasibility of the BP_3_ monolayer as an efficient and robust anode material, contributing to the development of cost-effective and high-performance SIBs.

## 2 Computational details

All first-principles calculations were carried out within the framework of density-functional theory (DFT), as implemented in the simulation package Quantum Espresso.^20^ The interaction between valence electrons and ionic cores was described by the projector-augmented-wave (PAW) method.^21^ Exchange–correlation effects were treated using the generalized-gradient approximation (GGA) in the Perdew–Burke–Ernzerhof (PBE) parametrization.^22^ A plane-wave kinetic-energy cutoff of 450 eV was employed for all calculations. The BP_3_ monolayer was modeled using a periodic supercell separated by a vacuum region of at least 18 Å along the out-of-plane direction to eliminate spurious interactions between periodically repeated images. The phonon dispersion was calculated using density functional perturbation theory (DFPT),^23^ as implemented in the Phonopy package, based on a 4 × 4 × 1 supercell. The Brillouin zone was sampled with a *Γ*-centered Monkhorst–Pack 12 × 12 × 1 *k*-point mesh. All geometries were fully relaxed until the residual forces on every atom were below 0.01 eV Å^−1^, while the electronic self-consistent loop was converged to 10^−8^ eV. To probe sodium-ion storage behaviour, one to several Na atoms were adsorbed at the most favourable sites identified from total-energy comparisons. Ion-migration pathways and diffusion barriers were obtained using the climbing-image nudged-elastic-band (CI-NEB) method^24^ with at least five intermediate images between the initial and final states; each image was relaxed until the perpendicular forces were smaller than 0.03 eV Å^−1^.

The adsorption energy (*E*_ad_) of Na atoms on the BP_3_ monolayer was calculated as:

1 *E*_ad_ = *E*_Na/BP_3__ − *E*_BP_3__ − *nE*_Na_,

where *E*_Na/BP_3__ is the total energy of the BP_3_ monolayer with *n* adsorbed Na atoms, *E*_BP_3__ is the total energy of the pristine BP_3_ monolayer, and *E*_Na_ is the energy per atom of bulk sodium. A negative value of *E*_ad_ indicates that the adsorption process is energetically favorable.

The open-circuit voltage (OCV) for Na insertion was evaluated from the total energy difference between two adjacent Na concentrations according to:2
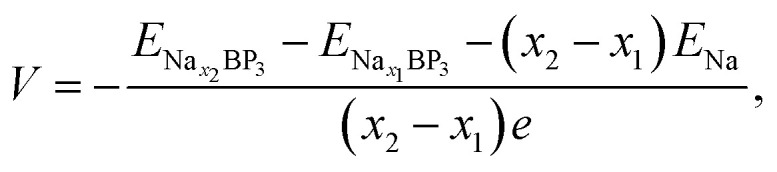
where *E*_Na_*x*_BP_3__ is the total energy of the Na_*x*_BP_3_ system, *E*_Na_ is the energy per atom of bulk bcc Na, *e* is the electron charge, and *x*_1_ and *x*_2_ denote the Na concentrations before and after insertion, respectively. A positive voltage value implies a thermodynamically favorable Na insertion reaction.

## 3 Results and discussion

**Fig. 1:**
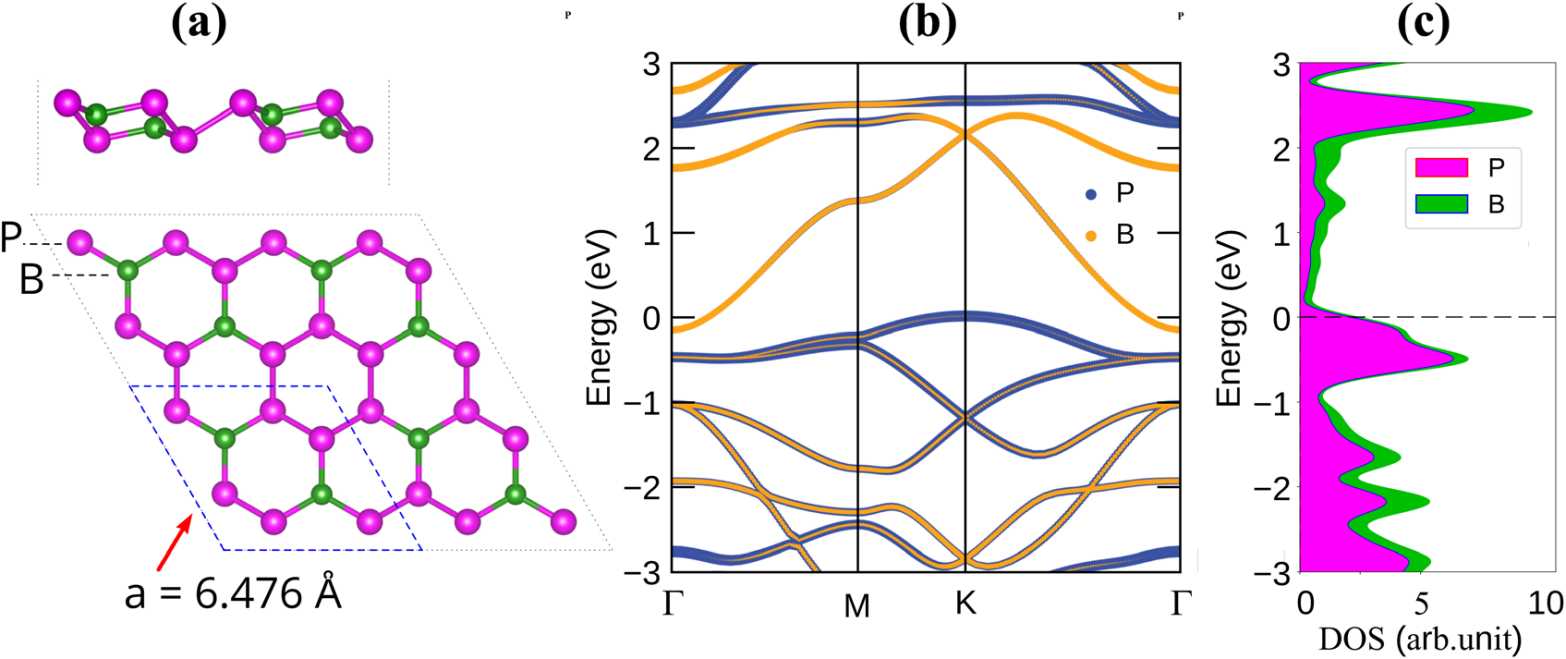
(a) Top and side views of the relaxed BP_3_ monolayer structure, (b) orbital-projected electronic band structure and (c) partial density of states (PDOS) of the BP_3_ monolayer. Boron (B) and phosphorus (P) atoms are represented by green and magenta spheres, respectively.

We began our investigation by analyzing the atomic and electronic structures of the BP_3_ monolayer. As shown in Fig. 1(a), the optimized BP_3_ monolayer adopts a puckered lattice with a distorted honeycomb motif, which qualitatively resembles the out-of-plane puckering feature of black phosphorene, although their symmetry and in-plane anisotropy are distinct. The primitive cell of the BP_3_ monolayer contains two boron (B) and six phosphorus (P) atoms. The optimized in-plane lattice constant was calculated to be 6.476 Å, forming an anisotropic layered framework. In this configuration, each B atom is covalently bonded to the surrounding P atoms, resulting in a robust and directionally extended 2D network. To further understand its electronic characteristics, we calculated the orbital-projected electronic band structure and PDOS, as shown in Fig. 1(b) and (c). The band structure indicates that the BP_3_ monolayer exhibits metallic behavior, as the valence and conduction bands overlap near the Fermi level along the Γ–M–K–Γ path. Notably, a significant hybridization between B *p* and P *p* orbitals occurs near the Fermi level, as evidenced by the overlapping contributions of B and P in both the band structure and the PDOS plots. This hybridization gives rise to delocalized electronic states that extend throughout the monolayer. The presence of such delocalized states crossing the Fermi level confirms the metallic nature of the BP_3_ monolayer. This intrinsic electrical conductivity is a highly advantageous feature for anode materials in SIBs, as it enables efficient electron transport during charge and discharge processes.

**Fig. 2:**
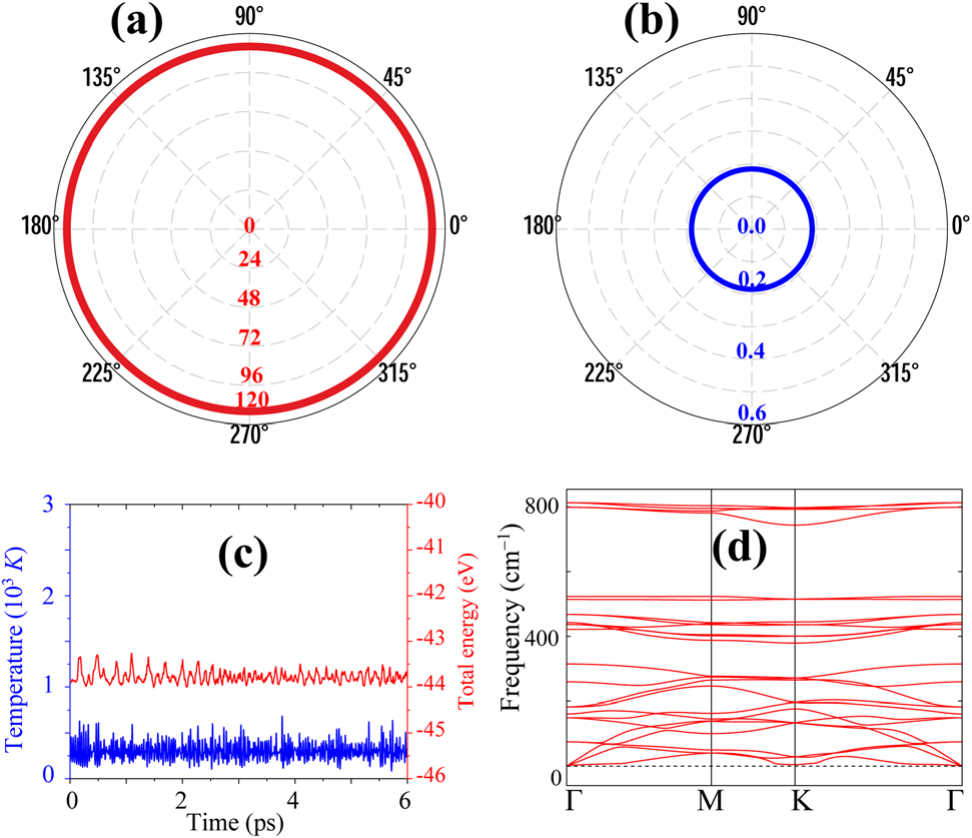
(a) The oriented dependence of Young's modulus, (b) Poisson ratio, (c) the fluctuations in temperature and total energy, and (d) phonon spectra of the BP_3_ monolayer.

To assess the mechanical robustness of the BP_3_ monolayer, we examined its elastic response within both anisotropic and polycrystalline frameworks. The polar plots of the Young's modulus and Poisson's ratio in Fig. 2(a and b) reveal perfectly circular contours, confirming complete in-plane isotropy. Quantitatively, the Young's modulus remains constant at 112.0 N m^−1^ in every direction, and the Poisson's ratio is uniformly 0.185. This stiffness surpasses or rivals that of recently reported 2D anodes—including BGe (56.7 N m^−1^),^25^ VS_2_ (98 N m^−1^), TiS_2_ (74 N m^−1^),^26^ BC_2_N (127 N m^−1^),^27^ and MoS_2_ (130 N m^−1^).^28,29^ The shear modulus is likewise direction-independent at 47.3 N m^−1^, yielding an anisotropy ratio of 1.0 for all elastic parameters. Averaged polycrystalline moduli, obtained *via* the Voigt–Reuss–Hill scheme, give a bulk modulus of 68.7 N m^−1^ and a bulk-to-shear ratio *K*/*G* = 1.45, indicative of balanced resistance to both volume and shape change. For a hexagonal 2D lattice, mechanical stability can be ensured by satisfying two independent Born–Huang criteria, namely *C*_11_ > 0 and *C*_11_ > |*C*_12_|.^30^ The calculated elastic constants of BP_3_ monolayer (*C*_11_ = 115.98 N m^−1^, *C*_12_ = 21.46 N m^−1^, and *C*_66_ = 47.26 N m^−1^) clearly fulfill these requirements. In addition, all principal eigenvalues of the stiffness tensor are positive (47.3, 94.5, and 137.4 N m^−1^, respectively), confirming that the elastic tensor is positive definite. These results consistently verify the mechanical stability and robustness of the BP_3_ monolayer.

Furthermore, to evaluate the thermal stability of the BP_3_ monolayer, *ab initio* molecular dynamics (AIMD) simulations were carried out at 300 K for a total simulation time of 6 ps with a time step of 1 fs. As shown in Fig. 2(c), both the system temperature and total energy exhibit only minor fluctuations throughout the simulation, without any abrupt changes or structural instabilities. The total energy remains nearly constant around −44 eV, and the temperature fluctuates slightly around the set point of 300 K. These results clearly demonstrate that the BP_3_ monolayer maintains its structural integrity under room-temperature conditions, thereby confirming its good thermal stability. In addition, the phonon dispersion of the BP_3_ monolayer (Fig. 2(d)) exhibits no imaginary frequencies throughout the Brillouin zone, confirming its dynamical stability.

**Fig. 3:**
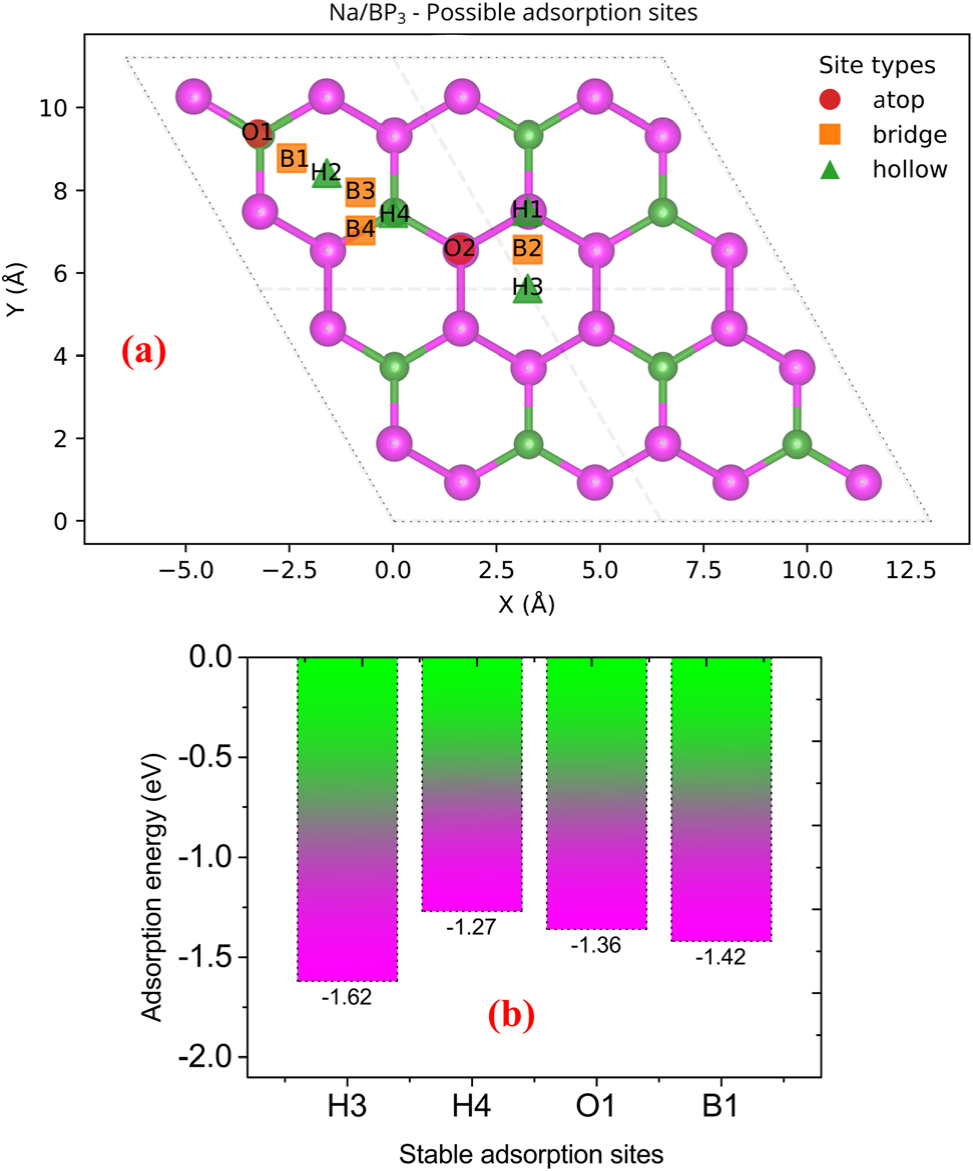
(a) Top view of the BP_3_ monolayer indicating ten high-symmetry Na adsorption positions, including atop (red circles), bridge (orange squares), and hollow (green triangles) sites. (b) Adsorption energies of the most stable Na adsorption configurations after structural relaxation: H3, H4, O1, and B1.

To evaluate the sodium storage capability of the BP_3_ monolayer, we systematically investigated ten symmetry-inequivalent Na adsorption positions, as shown in Fig. 3(a). These include two atop sites (O1 and O2), four bridge sites (B1–B4), and four hollow sites (H1–H4). All initial geometries were subjected to full structural relaxation to identify energetically favorable configurations. The results reveal that multiple initial placements converge to three unique adsorption states, demonstrating the dynamic nature of Na-surface interactions on the BP_3_ monolayer. Specifically, Na atoms initially positioned at H1, H3, B2, and O2—which all reside within the six-membered phosphorus hexagon—relax to a common hollow site denoted as H3. This site is located 1.357 Å above the BP_3_ monolayer and exhibits the strongest interaction with an adsorption energy of −1.62 eV. Similarly, Na atoms initially positioned at B3, B4, and H4 converge to a second hollow-type site labeled H4, located 1.349 Å above the monolayer, with a moderate binding energy of −1.27 eV. In contrast, the B1 and H2 sites relax to an atop-like configuration directly above a P atom (B1), located 1.904 Å from the surface and possessing an adsorption energy of −1.42 eV. Only the O1 site remains stable upon relaxation, retaining its initial atop configuration at 1.155 Å above the BP_3_ monolayer. It exhibits an adsorption energy of −1.36 eV, indicating favorable but weaker binding compared to the hollow-centered H3 configuration. As summarized in Fig. 3(b), four stable Na adsorption sites are identified: H3, H4, O1, and B1. Among them, the H3 site is the most thermodynamically favorable, suggesting that Na atoms preferentially occupy hollow regions within P-atom rings. The relatively strong adsorption energies (all below −1.2 eV) confirm the chemical affinity between Na and the BP_3_ monolayer, while the diversity of stable configurations provides flexibility for Na storage at varying coverages. These findings demonstrate the potential of the BP_3_ monolayer as a viable host material for Na-ion storage, where strong binding, geometric adaptability, and multiple stable adsorption sites could enhance the overall capacity and electrochemical reversibility.

**Fig. 4:**
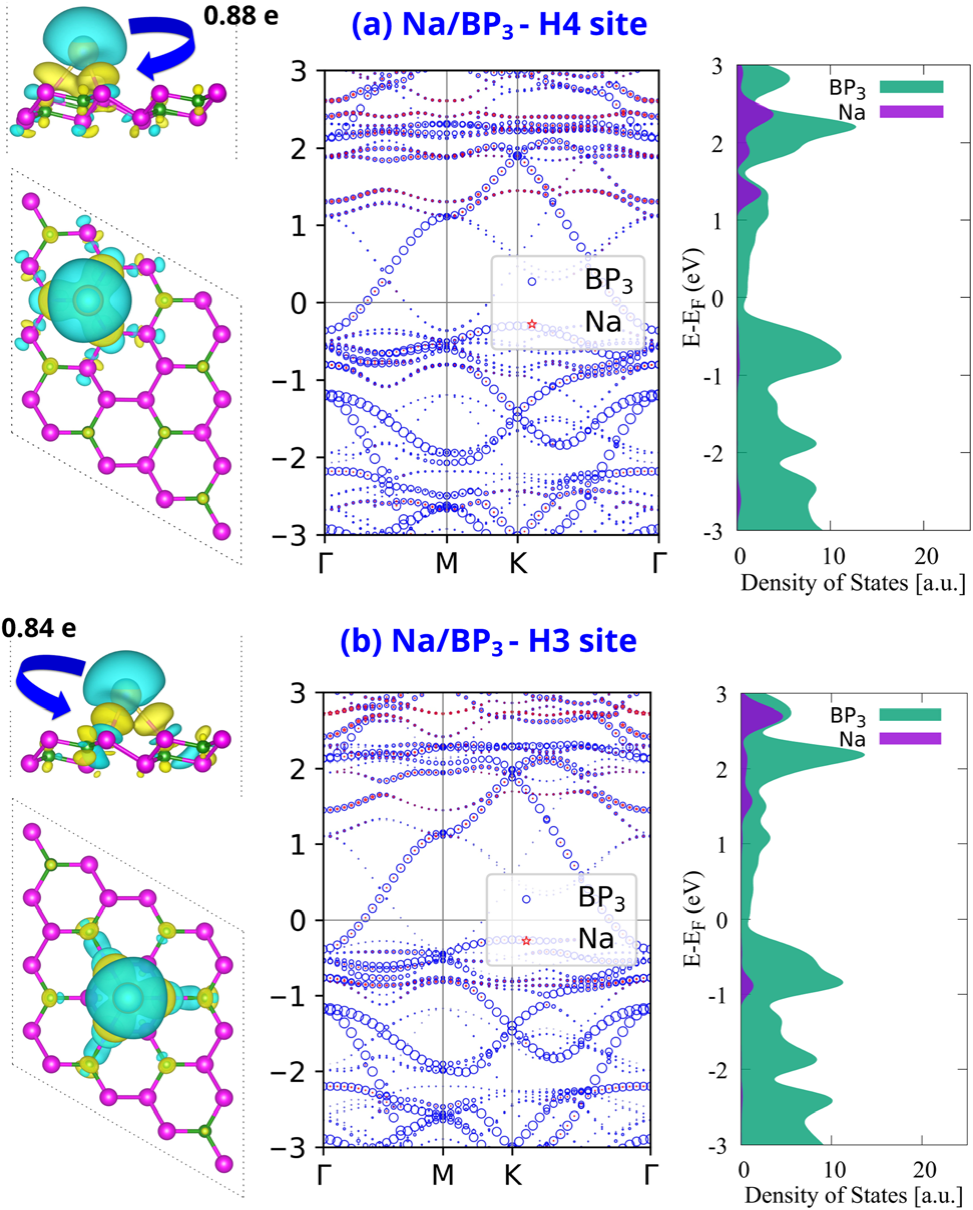
Charge density difference (CDD), weighted band unfolding, and partial density of states (PDOS) for Na adsorbed at the most stable adsorption site on the BP_3_ monolayer: (a) H4 site and (b) H3 site. In the CDD plots, yellow and cyan denote charge accumulation and depletion, respectively. The values next to the isosurface plots indicate the net charge transfer from Na to BP_3_ monolayer based on Bader charge analysis.

To gain deeper insight into the electronic interaction between Na ions and the BP_3_ monolayer, we analyze the charge density difference (CDD), weighted band unfolding, and projected density of states (PDOS) for two representative adsorption configurations: the H4 site and the H3 site, as shown in Fig. 4. The CDD maps in the left column of Fig. 4(a and b) reveal a pronounced charge redistribution upon Na adsorption. The CDD was calculated using the following expression:

3 Δ*ρ* = *ρ*_Na/BP_3__ − *ρ*_BP_3__ − *ρ*_Na_

where *ρ*_Na/BP_3__ is the charge density of the combined Na-adsorbed system, while *ρ*_BP_3__ and *ρ*_Na_ represent the charge densities of the pristine BP_3_ monolayer and the isolated Na atom, respectively, all computed in the same supercell.

In both H4 and H3 site configurations, the CDD plots show significant charge accumulation (yellow) around the interface between Na and the surface, and charge depletion (cyan) localized near the Na atom. This distribution clearly indicates a net charge transfer from Na to the BP_3_ monolayer. Quantitatively, Bader charge analysis reveals that the Na atom donates approximately 0.88*e* in the H4 site and 0.84*e* in the H3 site configurations. These values are significantly higher than the charge transferred from Na to the BP_2_ monolayer (only 0.41–0.44*e*), as reported by Ye *et al.*^17^ This strong charge-transfer capability suggests that BP_3_ possesses superior charge-accepting ability compared with BP_2_, which could enhance the electron/ion transport and improve the electrochemical activity. The middle panels display the weighted unfolded band structures. In both cases, the band dispersion of the BP_3_ monolayer is largely preserved, and the system maintains its metallic character. The Na-related bands exhibit negligible contribution near the Fermi level, confirming that Na acts mainly as a charge donor without introducing mid-gap states. This observation is further supported by the PDOS (right panel), where the electronic states around the Fermi level are dominated by the BP_3_ monolayer. The Na-derived states are shifted toward the conduction band, consistent with its electron-donating nature. The preservation of metallicity and absence of impurity-induced states at the Fermi level indicate that the Na/BP_3_ system remains conductive even after Na adsorption. Taken together, the results in Fig. 4 demonstrate that Na atoms strongly bind to the BP_3_ monolayer *via* substantial charge transfer while preserving its electronic conductivity—two key requirements for high-performance SIB anode materials.

**Fig. 5:**
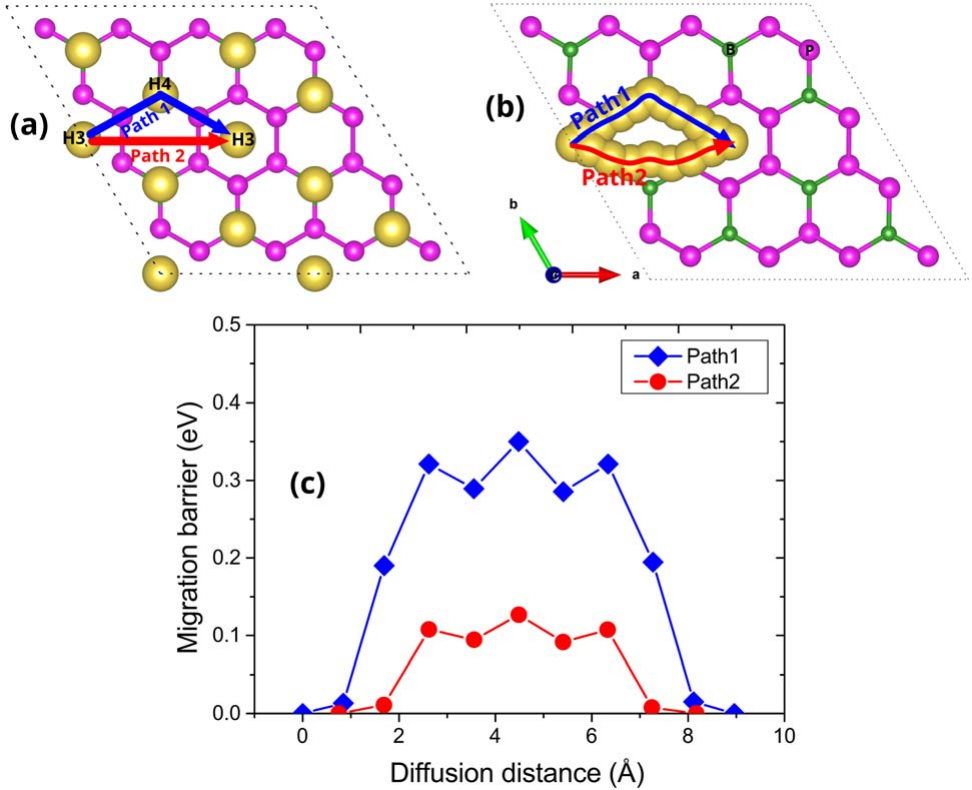
(a) Top and side views illustrating the possible Na-ion diffusion pathways on the BP_3_ monolayer, initially hypothesized based on the stable adsorption configurations. (b) Top and side views of the actual Na-ion diffusion trajectory obtained from CI-NEB calculations. (c) Calculated migration energy barriers along Path 1 (H3 → H4 → H3) and Path 2 (H3 → H3) based on the CI-NEB method.

To investigate the ion transport kinetics on the BP_3_ monolayer, we proposed two possible diffusion pathways for Na-ion migration, as shown in Fig. 5(a). The first pathway (Path 1) follows a two-step trajectory from an H3 site to a neighboring H3 site *via* an intermediate H4 position (H3 → H4 → H3). The second diffusion pathway (Path 2) was initially assumed to involve a direct hop between two adjacent H3 sites (H3 → H3). These diffusion paths were selected based on the distribution of Na atoms in the first adsorbed layer and the energetic favorability of the H3 site, as established in the previous adsorption energy analyses. However, upon performing CI-NEB calculations, we found that the direct H3–H3 jump in Path 2 is not dynamically favorable. Instead, during the NEB relaxation, the Na atom follows a slightly curved trajectory between the two H3 sites, spontaneously deviating toward an intermediate position located near the O1 site, as illustrated in Fig. 5(b). This deviation arises because the intermediate region provides a locally more stable configuration with a slightly lower potential energy compared to the direct route, causing the true minimum-energy pathway to bend toward this site. Therefore, the NEB-corrected Path 2 represents a more realistic and energetically optimized diffusion trajectory within the local lattice environment. The corresponding energy barriers for these diffusion processes are presented in Fig. 5(c). The lowest barrier of 0.13 eV is observed along the NEB-corrected Path 2, while the two-step Path 1 (H3 → H4 → H3) exhibits a higher maximum barrier of approximately 0.35 eV. This indicates that the diagonal H3–H3 route is kinetically more favorable for Na-ion migration. These low migration barriers, especially for the NEB-corrected Path 2, suggest that Na ions can diffuse efficiently across the BP_3_ monolayer. Such favorable ion mobility is essential for ensuring rapid charge/discharge capability, further supporting the viability of the BP_3_ monolayer as a high-performance anode material for sodium-ion batteries. For comparison, several other 2D anode materials have been reported with a wide range of Na-ion diffusion barriers, such as 0.03 eV for BP_2_,^17^ 0.07 eV for Janus WSSe,^31^ 0.11 eV for SiP_2_,^32^ 0.17 eV for SnC,^33^ 0.24 eV for BSi,^33^ and 0.73 eV for BC_3_N_3_.^34^ Although some 2D materials exhibit even lower diffusion barriers, the obtained value of 0.13 eV still remains the lowest reported for 2D anode systems and is sufficient to ensure fast Na-ion mobility within the BP_3_ monolayer.

**Fig. 6:**
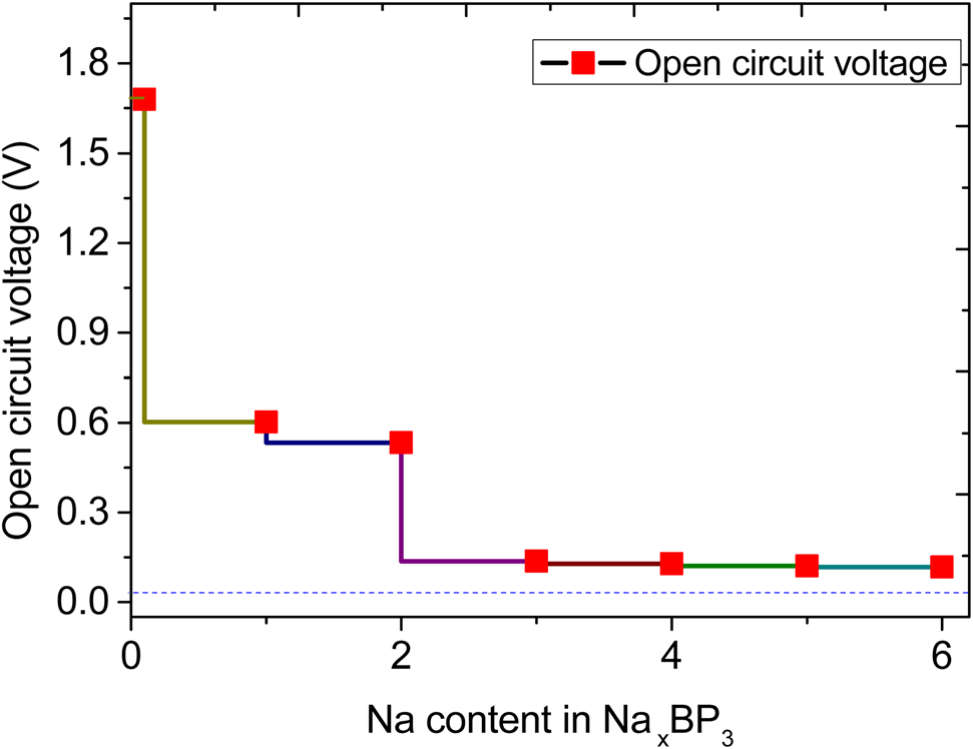
The open circuit voltage profiles for Na-ion adsorption on the BP_3_ monolayer with different concentrations of Na ions.

**Fig. 7:**
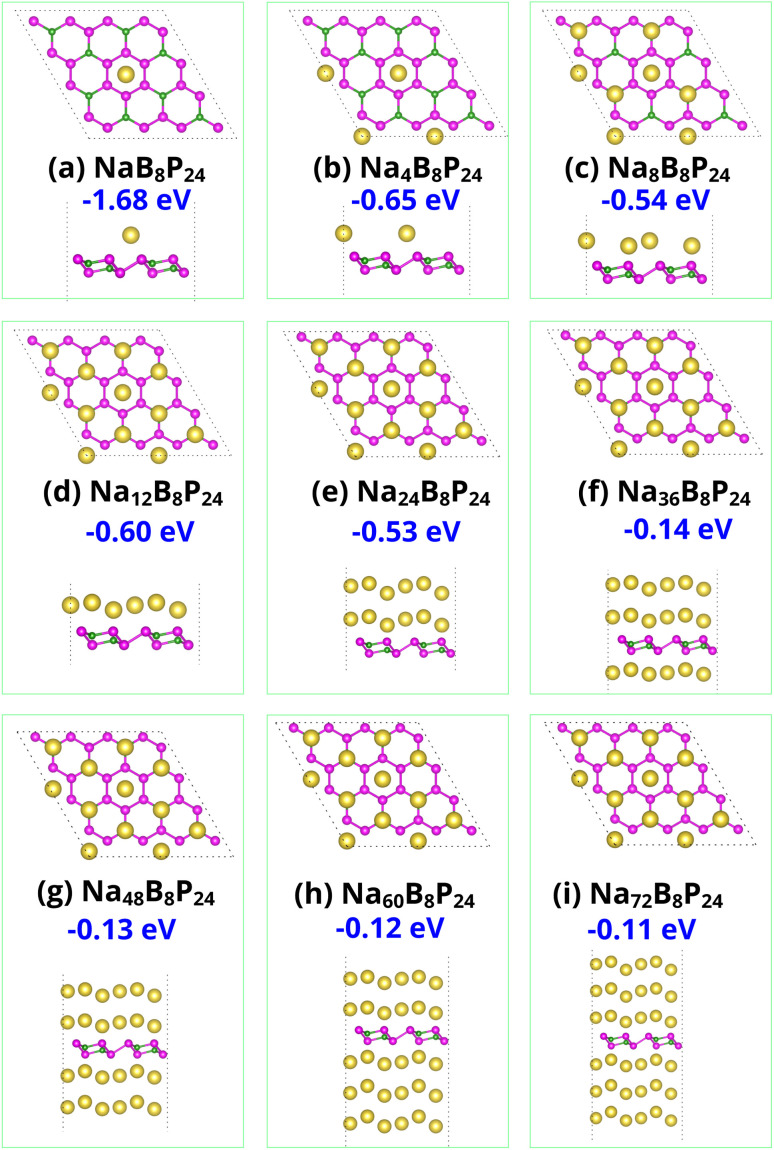
Top and side views of the Na-adsorbed BP_3_ monolayer at various sodiation levels, as shown in panels (a)–(i). Corresponding average adsorption energies (in eV) per Na atom are annotated below each optimized configuration.

Having identified the sequential Na adsorption configurations and favorable diffusion pathways, we now examine the open-circuit voltage (OCV) behavior and theoretical storage capacity of the BP_3_ monolayer with increasing Na content. The variation of OCV as a function of Na concentration (*x* in Na_*x*_BP_3_) is presented in Fig. 6. Initially, a remarkably high voltage of approximately 1.73 V is observed at low Na concentrations (*x* = 0.167), which corresponds to the strong binding at the most energetically favorable H3 sites (Fig. 7(a and b)). This abnormally high OCV can be interpreted from a thermodynamic perspective: at the early stage of sodiation, the first Na atoms preferentially occupy the most stable adsorption sites with the strongest binding affinity, leading to a substantial decrease in system energy and, consequently, a sharp voltage rise. Once these highly stable sites are filled, subsequent Na adsorption must occur at less favorable sites, where Na–Na repulsion and local lattice distortion effects begin to emerge, reducing the adsorption energy significantly. As a result, the OCV rapidly decreases to around 0.60 V for *x* = 1 and gradually declines to 0.53 V as *x* increases to 2, reflecting the progressive occupation of weaker adsorption sites such as H4 (Fig. 7(c and d)). When the Na concentration further increases from 2 to 3, the OCV drops to about 0.14 V and eventually approaches a nearly constant low-voltage region (0.13–0.11 V) for multilayer Na configurations (*x* = 3–6). This quasi-plateau behavior corresponds well with the weak binding energies shown in Fig. 7(h–i), where additional Na layers form above the first adsorbed layer. The sharp initial OCV increase and its subsequent drop thus reflect a phase-like transition between the pristine and dilute intercalation states near *x* ≈ 0.167, underscoring the decisive role of the first adsorption sites in shaping the overall voltage–capacity characteristics of BP_3_ as a Na-ion anode material. The average OCV of Na/BP_3_ was calculated to be 0.27 V, which compares favorably with those of other 2D monolayers upon Na adsorption, including PC_6_ (0.40 V),^35^ SnSe_2_ (0.66 V),^36^ graphene-like AlP_3_ (0.28 V),^37^ and tetragonal BN monolayer (0.35 V).^38^ These results suggest that the BP_3_ monolayer possesses a relatively low and stable average voltage, which is advantageous for achieving high energy density and favorable compatibility with conventional electrolytes in sodium-ion batteries.

From the complete Na adsorption configuration Na_72_B_8_P_24_ (Fig. 7(i)), which involves six Na layers symmetrically adsorbed on both sides of the monolayer, we estimated the theoretical storage capacity of the BP_3_ monolayer. The specific capacity *C* was calculated using:4
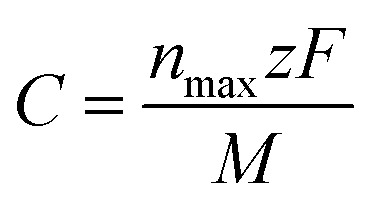
Here, *n*_max_ = 72 is the maximum number of adsorbed Na ions, *z* = 1 is the charge number per Na ion, *F* = 26.801 Ah mol^−1^ is the Faraday constant, and *M* is the molar mass of the pristine BP_3_ supercell. Substituting the computed values, the theoretical specific capacity of the BP_3_ monolayer was determined to be 2325.58 mAh g^−1^, which ranks among the highest ever reported for 2D anode materials in SIBs. This value significantly surpasses most known 2D materials. For instance, conventional anode candidates such as MXene Ti_3_C_2_T_*x*_, Mo_2_CO_2_, and Janus MoSSe monolayers deliver relatively low capacities of 125, 379, and 510 mAh g^−1^, respectively.^39–41^ Even several recently proposed materials with promising characteristics exhibit inferior capacities. The C_6_BN monolayer offers a Na capacity of 553 mAh g^−1^,^42^ while the SiC_2_ monolayer provides 1203 mAh g^−1^.^43^ Monolayers BSi and C_6_B_4_ exhibit improved capacities of 1034 and 1395 mAh g^−1^, respectively,^44,45^ and even the Dirac TiC monolayer reaches an energy density of 2015 mWh g^−1^ for Na.^46^ Phosphorus-based materials such as BP and BP_2_ monolayer – discussed in the Introduction section – exhibit favorable electrochemical properties, yet their Na storage capacities remain substantially lower than that of the BP_3_ monolayer. Notably, even the high-capacity B_3_P monolayer, with a value of 1691 mAh g^−1^,^18^ is still outperformed by the BP_3_ monolayer. The exceptional capacity of the BP_3_ monolayer arises from its ability to accommodate dense Na layers on both sides of the puckered surface while maintaining favorable adsorption energies. In addition, the system retains metallic conductivity after sodiation and exhibits strong Na-surface interactions, ensuring rapid ion diffusion and efficient electron transport. These synergistic features make the BP_3_ monolayer a highly competitive and next-generation anode material for SIBs targeting ultrahigh energy density.

To further evaluate the structural robustness of BP_3_ during sodiation, we monitored both the intrinsic volume change of the host framework (considering only B/P atoms within the monolayer) and the effective electrode volume (including the adsorbed Na layers on both sides). Since the in-plane lattice constants (*a* and *b*) of the BP_3_ monolayer remain nearly constant during the sodiation process, the relative volumetric expansion was directly evaluated from the change in intrinsic and effective layer thicknesses according to:5
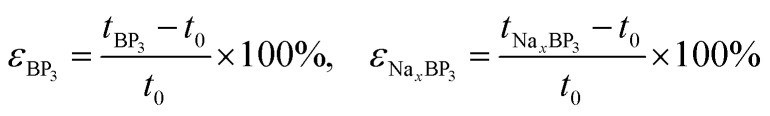
where *t*_0_ denotes the pristine layer thickness of BP_3_ and *t*_BP_3__ and *t*_Na_*x*_BP_3__ are the intrinsic and effective thicknesses during Na intercalation, respectively.

As summarized in Table 1, the intrinsic volume expansion of the BP_3_ host framework remains very small, ranging from −1.93% to +3.98% throughout the entire sodiation process. Such negligible expansion confirms the mechanical resilience of the puckered BP_3_ lattice, indicating that the host structure can accommodate Na insertion without significant distortion or collapse. In contrast, the effective electrode thickness—comprising both the host framework and the external Na layers—increases substantially, reaching ∼1600% when fully covered by Na. However, this remarkable expansion originates not from swelling of the BP_3_ lattice but from the progressive stacking of Na atoms on both sides of the monolayer surface. Therefore, although the overall electrode experiences a large apparent size increase due to external Na accumulation, the intrinsic structural integrity of BP_3_ remains well preserved. These findings highlight two key aspects: (i) BP_3_ provides an exceptionally stable host lattice with very low internal stress, suggesting good reversibility and high cycling durability, and (ii) while the high theoretical capacity (2325.58 mA h g^−1^) entails substantial effective expansion that could challenge practical applications—such as the reduced volumetric energy density and interfacial stress with the electrolyte—this is a general issue for high-capacity anodes rather than a specific drawback of BP_3_. Engineering strategies such as functionalized binders^47^ or porous electrode design^48^ could mitigate this effective expansion while fully exploiting the outstanding capacity and intrinsic structural stability of BP_3_.

**Table 1:** Variation of the intrinsic thickness (*t*_BP_3__), effective thickness (*t*_Na_*x*_BP_3__), and corresponding relative volumetric expansion (*ε*_BP_3__ and *ε*_Na_*x*_BP_3__) of the BP_3_ monolayer during Na intercalation. Here, *x* denotes the number of intercalated Na layers; length is given in Å, and volumetric expansion is expressed in percent (%)

*x*	*t*_BP_3__	*t*_Na_*x*_BP_3__	*ε*_BP_3__	*ε*_Na_*x*_BP_3__
0	1.16	1.16	0	0
1	1.19	3.71	2.60	219
2	1.14	6.04	−1.93	419
3	1.17	9.42	0.78	710
4	1.20	12.88	2.79	1007
5	1.21	16.30	3.98	1301
6	1.21	19.77	3.98	1600

**Fig. 8:**
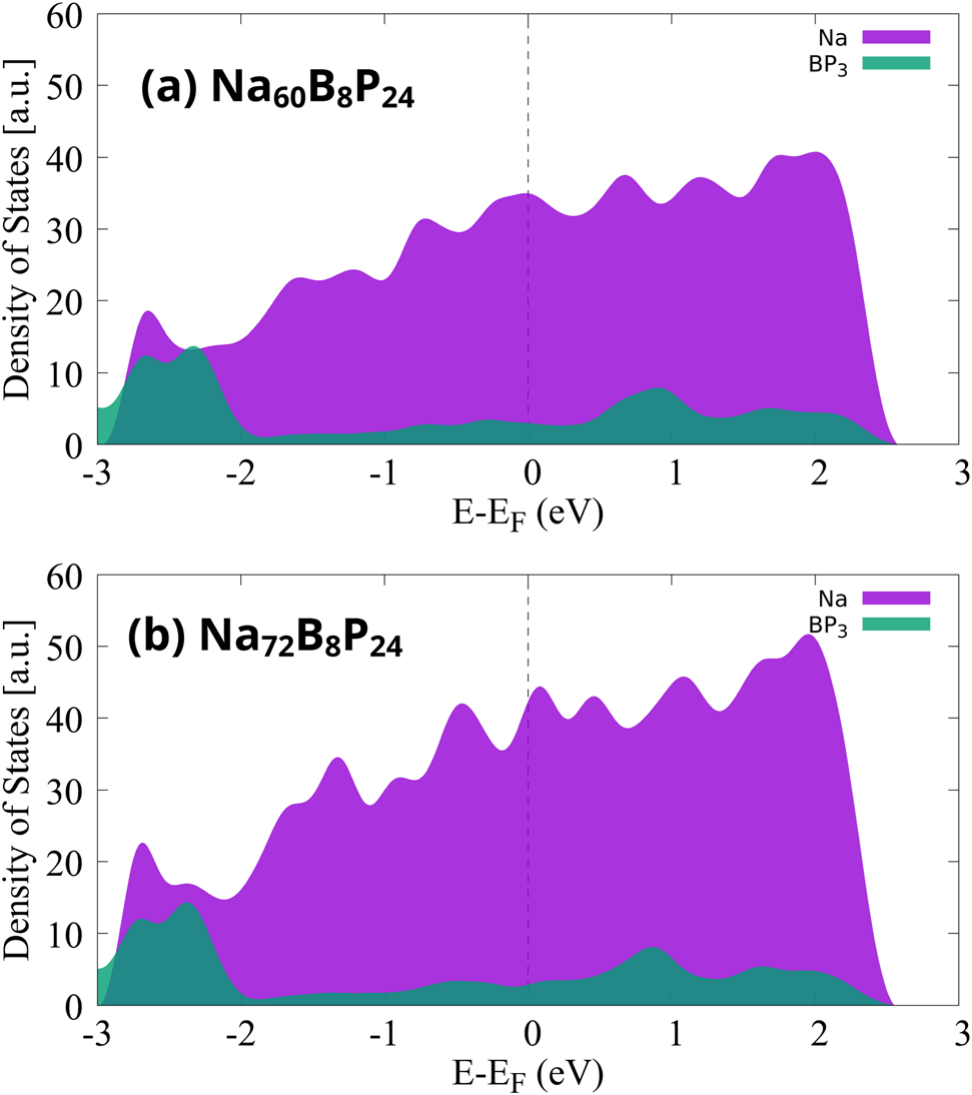
The contributions of the Na atom and BP_3_ monolayer for different Na concentrations of (a) Na_60_B_8_P_24_ and (b) Na_72_B_8_P_24_.

Finally, to assess whether the metallic conductivity of the BP_3_ monolayer is preserved after extensive sodiation, we analyzed the partial density of states for the highly sodiated configurations Na_60_B_8_P_24_ and Na_72_B_8_P_24_, as illustrated in Fig. 8(a and b). In both cases, the Fermi level lies well within a continuum of states, confirming that these systems remain metallic even at high Na concentrations. The dominant contribution to the states near the Fermi level originates from the Na atoms (in purple), with a smaller contribution from the BP_3_ monolayer (in green). This behavior indicates that the adsorbed Na layers not only retain but also enhance the overall electrical conductivity of the sodiated system. Such metallicity ensures efficient electron transport throughout the charge/discharge process, a critical requirement for high-performance anode materials.

## 4 Conclusion

In summary, we have conducted a comprehensive DFT-based investigation of the BP_3_ monolayer, aiming to assess its viability as an anode material for sodium-ion batteries. Our results reveal that the BP3 monolayer exhibits excellent mechanical robustness and thermal stability, along with intrinsic metallic conductivity. Na^+^ diffusion on the surface proceeds with remarkably low energy barriers, with the most favorable migration pathway requiring only 0.13 eV, suggesting rapid charge transport kinetics. Upon full sodiation, the system evolves into Na_72_B_8_P_24_, achieving an ultrahigh theoretical capacity of 2325.58 mAh g^−1^—placing it among the top-performing 2D anode materials predicted to date. Importantly, the metallic nature of BP_3_ is preserved throughout all sodiation stages, ensuring continuous electronic conductivity during cycling. These exceptional electrochemical characteristics position the BP_3_ monolayer as a highly promising candidate for next-generation sodium-ion batteries. Overall, our results provide a solid theoretical foundation that serves as a valuable reference for future experimental synthesis and practical evaluation of BP_3_-based Na-ion anodes.

## Conflicts of interest

There are no conflicts of interest to declare.

## Data Availability

All essential data are presented in the manuscript. Large raw computational files are available upon reasonable request.
